# Supplementary figures and images for: Patterns of belatacept use and risk of post-transplant lymphoproliferative disorder in US kidney transplant recipients: An analysis of the Organ Procurement and Transplantation Network database
Source: PLoS One. 2025 Jan 10;20(1):e0311935. doi: 10.1371/journal.pone.0311935 (PMC11723631; doi:10.1371/journal.pone.0311935)

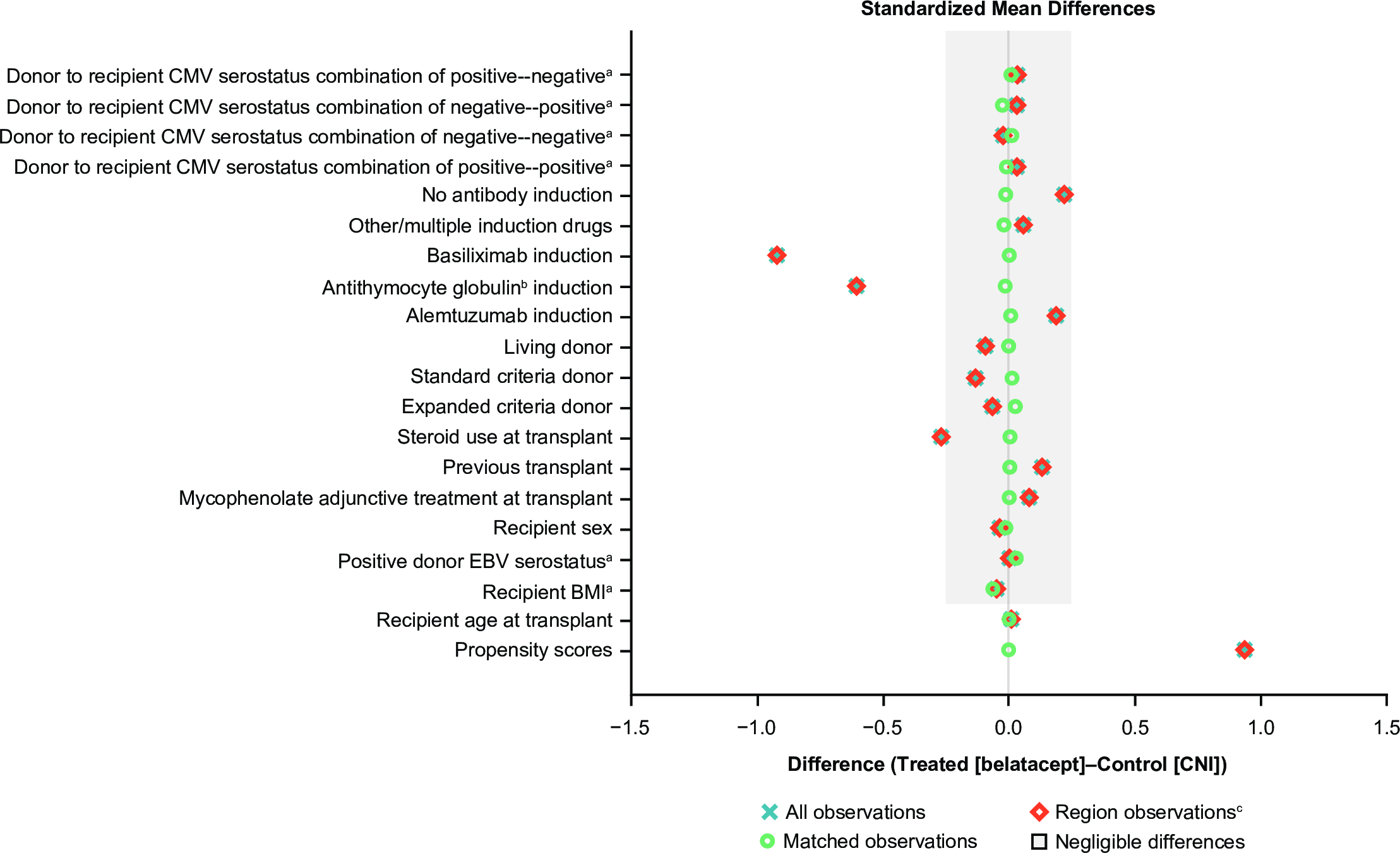

Supplement: S1 Fig — aUsing imputed value for missing. bRefers to rabbit-derived antithymocyte globulin (Thymoglobulin). c“Region” refers to observations with overlapping propensity scores for the treated (belatacept) and control (CNI) groups. BMI, body mass index; CMV, cytomegalovirus; CNI, calcineurin inhibitor; EBV, Epstein-Barr virus. (TIF) [file pone.0311935.s001.tif]

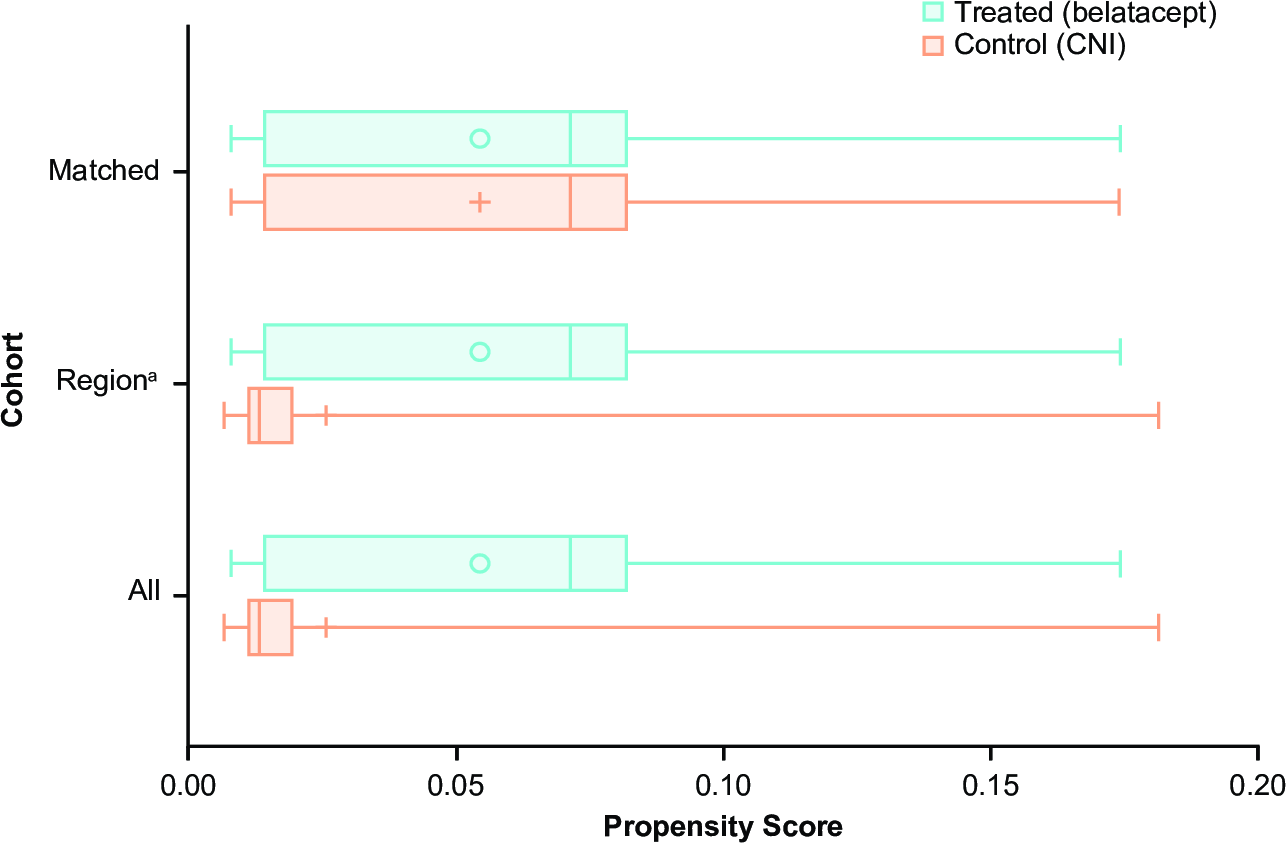

Supplement: S2 Fig — a“Region” refers to observations with overlapping propensity scores for the treated (belatacept) and control (CNI) groups. (TIF) [file pone.0311935.s002.tif]
