# Supplementary material for: Patterns of belatacept use and risk of post-transplant lymphoproliferative disorder in US kidney transplant recipients: An analysis of the Organ Procurement and Transplantation Network database
Source: PLoS One. 2025 Jan 10;20(1):e0311935. doi: 10.1371/journal.pone.0311935 (PMC11723631; doi:10.1371/journal.pone.0311935)
Supplement: S1 Table — (PDF) [file pone.0311935.s003.pdf]

S1 Table. Demographic and clinical characteristics in the matched cohort.

Characteristics	Belatacept (n = 1631)	CNI (n = 1631)	p-value
Age at transplant (years)			0.750
Mean (SD)	51.9 (13.7)	51.9 (14.1)	
Median (IQR)	53.0 (42.0−63.0)	54.0 (43.0−63.0)	
Sex, n (%)			0.691
Female	613 (37.6%)	624 (38.3%)	
Male	1018 (62.4%)	1007 (61.7%)	
Body weight (kg)			0.716
Mean (SD)	82.3 (18.9)	81.9 (19.2)	
Median (IQR)	81.0 (68.0−95.3)	80.5 (68.4−94.5)	
BMI (kg/m^2)			0.140
Mean (SD)	27.8 (5.2)	28.2 (5.6)	
Median (IQR)	27.6 (23.8−31.8)	27.9 (24.0−32.0)	
Previous transplant, n (%)			0.951
No	1489 (91.3%)	1488 (91.2%)	
Yes	142 (8.7%)	143 (8.8%)	
Donor type, n (%)			0.806
Expanded criteria donor	190 (11.6%)	202 (12.4%)	
Living donor	622 (38.1%)	620 (38.0%)	
Standard criteria donor	819 (50.2%)	809 (49.6%)	
Donor EBV serostatus, n (%)			0.402
Negative	29 (1.8%)	23 (1.4%)	
Positive	1602 (98.2%)	1608 (98.6%)	
Type of antibody induction, n (%)			0.931
Alemtuzumab	155 (9.5%)	158 (9.7%)	
Basiliximab	971 (59.5%)	971 (59.5%)	
Antithymocyte globulin[a]	373 (22.9%)	383 (23.5%)	
Other/multiple drugs[b]	60 (3.7%)	53 (3.2%)	
No induction	72 (4.4%)	66 (4.0%)	
Donor-recipient CMV serostatus, n (%)			0.889
Negative-negative	280 (17.2%)	286 (17.5%)	
Negative-positive	384 (23.5%)	365 (22.4%)	
Positive-negative	258 (15.8%)	262 (16.1%)	
Positive-positive	709 (43.5%)	718 (44.0%)	
Mycophenolate adjunctive treatment, n (%)			1.000
No	88 (5.4%)	88 (5.4%)	
Yes	1543 (94.6%)	1543 (94.6%)	
Steroid use at transplant, n (%)			0.929
No	317 (19.4%)	315 (19.3%)	
Yes	1314 (80.6%)	1316 (80.7%)	

The distribution of characteristics in the belatacept and CNI treatment groups were compared using the Pearson chi-square test for categorical variables and Kruskal-Wallis test for continuous variables.

[a]Refers to rabbit-derived antithymocyte globulin.

[b]Included equine-derived antithymocyte globulin, muromonab-CD3, or multiple drugs.

BMI, body mass index; CMV, cytomegalovirus; CNI, calcineurin inhibitor; EBV, Epstein-Barr virus; IQR, interquartile range; SD, standard deviation.
