# Supplementary material for: Patterns of belatacept use and risk of post-transplant lymphoproliferative disorder in US kidney transplant recipients: An analysis of the Organ Procurement and Transplantation Network database
Source: PLoS One. 2025 Jan 10;20(1):e0311935. doi: 10.1371/journal.pone.0311935 (PMC11723631; doi:10.1371/journal.pone.0311935)
Supplement: Attachment — Submitted filename: Response to Reviewers1.docx [file pone.0311935.s004.docx]

| **Comments** | **Response and Changes Made** | **Page Numbers**  **(tracked draft)** |
| --- | --- | --- |
| **Reviewer 1** | | |
| Thank you for allowing me to review this manuscript for your journal. The authors present a long awaited analysis of the incidence of PTLD in patients receiving belatacept versus those receiving a CNI. The authors did a wonderful job with the statistics and reporting and should be commended. I have a few comments to strengthen the manuscript. | We thank the reviewer for their comments. | NA |
| 1. The authors need to make it more clear how they "know" patients are on belatacept or CNI throughout the 5 year period. In the methods, it is indicated that the TRR and TRF forms are utilized. These forms are routinely inaccurate. It would be an important addition to the manuscript to describe this in more detail. The results hinge on the confidence level of the data included. I am concerned that some patients started belatacept and stopped early and/or others started and stopped CNI at unknown time periods making the inclusion into different groups problematic. | We used an intent-to-treat analytic approach in the study, where patients who received belatacept and/or CNI at transplant per their TRR records were included in the belatacept group, regardless of what they received on 1-year follow-up records. The OPTN database only captures immunosuppression at transplant and on the 1-year TRF records. The majority (84%) of recipients in our study cohort who started on belatacept at discharge stayed on belatacept at 1 year. We have added language to the manuscript Methods (new text highlighted below) to clarify the intent-to-treat approach:  “An intent-to-treat approach was used in the analyses (i.e., any report of belatacept or CNI use on the TRR records, regardless of any switches that may have occurred thereafter), and individuals who received both belatacept and CNI were included in the belatacept treatment group.”  We understand that there is a possibility of inaccurate information submitted to the OPTN registry, which is a limitation of large-scale registries. In an attempt to improve the accuracy of the TRR and TRF forms used in the study, the current analyses were limited to validated records to account for a 2-month lag that transplant centers may have in data submission to the OPTN registry database, to ensure that the transplant centers have finished completing the forms accurately and all the records have passed the appropriate validation and error checks. | 5–6 |
| 2. The authors should include how long patients were on each therapy if known. | Unfortunately, duration of immunosuppression is not collected in OPTN database. Additionally, the OPTN database only captures immunosuppression at transplant and 1-year post-transplant. |  |
| 3. There may be a typo on page 13, “PTLD histopathology was reported in 8.9% of belatacept-treated”…". Should this instead be 89%? | We thank the reviewer for pointing out this error and have corrected the percentages as follows:  “PTLD histopathology was reported in 88.9% of belatacept-treated and 89.~~1~~3% of CNI-treated patients who developed PTLD.” | 15 |
| 4. The paragraph on page 9 describing the baseline characteristics is somewhat clunky and not clearly written. It might be better instead to just refer the reader to table 1 and avoid redundancy. | We have removed the following text from the manuscript Results:  “~~Compared with transplant recipients in the CNI treatment group, those in the belatacept treatment group were less likely to have received a standard criteria donor kidney (p < 0.0001), more likely to have received steroid therapy at transplant (p < 0.0001), and more likely to have experienced acute rejection within 1 year post-transplant (p < 0.0001). In addition, belatacept-treated transplant recipients were less likely to have received mycophenolate as adjunctive treatment (p = 0.0005) and more likely (p < 0.0001) to have received a previous transplant, although the proportional differences between treatment groups for these two parameters were small (≤4%) and possibly not of clinical significance. The type of induction therapy also differed between the two treatment groups (p < 0.0001), with anti-thymocyte globulin and basiliximab being used less and more frequently, respectively, in the belatacept treatment group.~~” | 11 |
| **Reviewer 2** | | |
| This study attempted to compare the rates of PTLD in adult EBV +ve kidney transplant recipients who have received belatacept vs CNI between Jun 15 2011 to Jun 14 2016 using OPTN database. It's difficult to draw strong conclusions given the confounding variables, the small # of events in both groups and short duration of follow-up. | We thank the reviewer for their comments. We agree that we are limited by the relatively small number of PTLD cases in the study cohort. However, this study did include all transplant recipients in the US from the OPTN database, and we believe it would still provide valuable real-world information on PTLD incidence for the belatacept and CNI groups during the study period. We have acknowledged the sample size limitations in the Discussion:  “Among EBV-seropositive, kidney-only transplant recipients in the present analysis, the 5-year Kaplan–Meier incidence rate of PTLD was low, regardless of whether patients received belatacept or CNI at transplant (0.70% and 0.48%, respectively).”  “…~~only nine cases of PTLD were recorded among belatacept-treated patients, which~~ the low number of PTLD events in both the belatacept and CNI groups (nine and 225 cases, respectively) limited the types of statistical analyses that could be performed; results should be interpreted with caution.”  “…Among adult, EBV-seropositive, kidney-only transplant recipients receiving belatacept ~~in routine clinical practice~~ included in this study, the risk of PTLD remained relatively low, that is, comparable to that observed among patients receiving CNI-based immunosuppression and similar to that in the belatacept clinical registration trials.” | NA  19  21  21 |
| 1. The following confounders will increase the risk of PTLD and would suggest including some discussions in the limitations:   In the overall cohort,   a) The induction agents used were very different between the two groups favoring belatacept: Antithymocyte globulin (ATG) was used in 50.8% vs 22.9% and campath 15.7% vs 9.5% while basiliximab 59.5% vs 18.7% in CNI vs belatacept group, respectively (Table 1) b) Mean/median doses of ATG and Campath used in each group, if available c) Rejection rate at 1 year 22.3% vs 9.5% in belatacept vs CNI group - while it's true that maintenance immunosuppressants would usually be intensified, this is rejection rate at 1 year and it's unclear of rejection rates after 1 year. Also, if pts were switched from CNI to belatacept, the risk of rejection would be higher during the switchover period (this may also explain why half of the belatacept pts were also taking CNI at the same time) d) What were the rejection rates during the entire study period? Patients were recruited between 2011 to 2015 and followed until 2019. e) Half of the belatacept group was also taking CNI with 3 PTLD cases in this combo group. I assume the combination group was included in the belatacept group to increase its sample size. Were these patients being transitioned off CNI? f) Duration of follow-up in each group was unclear. Table 2 - were all pts followed for 5 years if last patient was enrolled in Jun 2016 and study closed in Apr 2019. What was the mean/median duration of follow-up in each group? g) While most PTLD cases surfaced early post-transplant, the LONG TERM PTLD risk of belatacept is unknown as BENEFIT and BENEFIT-EXT were relatively short duration. It would be more interesting to follow these patients for a longer period of time to realize the true PTLD risk of belatacept. | a) Given the approved labeling, the difference in induction agent was expected. Differences in ATG and basiliximab induction between the belatacept and CNI groups, as well as other relevant characteristics, were taken into account in the propensity score matching analysis. As shown in Table S1, there was a similar distribution of induction types between belatacept and CNI in the matched cohort. In the matched cohort, the difference in PTLD incidence between the belatacept and CNI groups did not reach statistical significance. This is noted in the Discussion:   "…to account for differences in baseline covariates, a PSM multivariable analysis was undertaken. Data from the matched cohort confirmed the findings of the overall study population; there were no statistically significant differences between risks of PTLD in belatacept- and CNI-treated patients, regardless of whether PTLD incidence rates were calculated based on patient-years at risk or using the Kaplan–Meier method.”   b) Dosage is not collected in OPTN database. c) We have added switchover from CNI to belatacept as a potential confounder to the Discussion (see below). d) The overall 1-year treated acute rejection rate was 9.9% in the study cohort. Unfortunately, we did not have access to treated acute rejection information beyond 1 year post-transplant. e) With the intent-to-treat analytic approach, we categorized recipients with belatacept and CNI into the belatacept group, regardless of switches thereafter. Of those who were reported with both belatacept and CNI at transplant and were alive with a functioning graft at 1 year, 281 (40%) were transitioned off of CNI by 1 year post-transplant. f) The median duration of follow-up was almost 4 years in both groups (1389 days in belatacept group and 1436 days in CNI group). These data have now been added to the Results section:   “The median duration of follow-up was 1389 days in the belatacept group and 1436 days in CNI group.”   g) We agree with the reviewer that longer-term risk of PTLD with belatacept use would be of great interest, which could potentially be captured in a future study. We have added this as a limitation to the Discussion (see below).   New text has been added to the Discussion to address items b, c, e, and g as limitations:  “Some variables that could potentially impact the risk of PTLD are not specifically captured in the OPTN database, such as dosing frequency, details of switchover from CNI to belatacept, or immunosuppression usage beyond 1 year; data collection of comorbidities at transplant is also limited.”  “…Finally, our study was limited to PTLD risk within 5 years of transplant. Future studies would potentially be needed to assess the longer-term risk of PTLD from belatacept usage.” | 20  8  21  21 |
| 2. Table 3 PTLD cases - 47.6% of CNI group received other/multiple drugs vs 0% in the belatacept group. What were these agents and could these have influenced the PTLD risks? | We thank the reviewer for pointing this out. After carefully checking the data, we realized there were transcriptions errors in Table 3. There were only eight CNI recipients (3.6%) with PTLD that were reported with other/multiple drugs – of these, six were reported with both Thymoglobulin and Simulect, one was reported with Campath and Simulect, and one was reported with Atgam. We have corrected Table 3 and added a footnote (text shown below) to clarify the drugs used by patients in the “Other/multiple drugs” category. We apologize for this oversight.  “^e^Included patients treated with equine-derived antithymocyte globulin (n = 1), rabbit-derived antithymocyte globulin and basiliximab (n = 6), or alemtuzumab and basiliximab (n = 1).” | 14  15 |
| 3. S1. Why are ATG & 1-yr rejection rates not included in the model? Of the covariates used for matching, these are likely more relevant to the outcome of interest. Please explain 'region observations', 'all observations' | The reason we did not include 1-year treated acute rejection was because the focus of the current analysis was to look at baseline characteristics at transplant. If we used 1-year rejection as a risk factor, we would have to condition the analysis to those surviving to 1 year since we would be using a post-transplant outcome to assess another post-transplant outcome. With the limited number of PTLD cases that we had, we decided to limit our analysis to baseline characteristics.  ATG induction was considered in the propensity score matched analysis – the reason it did not appear in Fig S1 is because it was treated as the baseline induction group in the PSM model and was not included in Fig S1 by default. For clarity and consistency, we have updated Fig S1 to show ATG as well as other baseline groups used for the categorical variables.  To clarify the use of “Region” in the Fig S1, we have added the following footnote to the figure:  “^c^“Region” refers to observations with overlapping propensity scores for the treated (belatacept) and control (CNI) groups.” | 29 |
| 4. S2 Fig is difficult to understand. Please label the y-axis and legend accordingly. please explain 'region', 'observations', Control=CNI, treated = belatacept | We have made clarifying edits to Fig S2, including a change from “Observations” to “Cohort” in the y-axis label and the addition of a footnote to define “region”, as shown below:  “^a^“Region” refers to observations with overlapping propensity scores for the treated (belatacept) and control (CNI) groups.” | 29 |
| 5. Event rates were low in both overall and PSM cohorts - this could explain why no statistically significant differences were found between belatacept and CNI groups. I'm not sure if we can conclude 'the risk of PTLD is comparable to that observed among pts receiving CNI-based immunosuppression' (p.19) | We have edited the text in the Discussion to capture the limitation of the low number of PTLD events:  “…~~only nine cases of PTLD were recorded among belatacept-treated patients, which~~ the low number of PTLD events in both the belatacept and CNI groups (nine and 225 cases, respectively) limited the types of statistical analyses that could be performed; results should be interpreted with caution.”  “…Among adult, EBV-seropositive, kidney-only transplant recipients receiving belatacept ~~in routine clinical practice~~ included in this study, the risk of PTLD remained relatively low, that is, comparable to that observed among patients receiving CNI-based immunosuppression, and similar to that in the belatacept clinical registration trials.” | 21  21 |
| 6. Discussions - seems biased to me just focusing on confounders that biased against belatacept (p.17-18); underreporting of PTLD in CNI treated patients (p.19). Were all PTLD cases reported in belatacept group and only CNI group was underreported? | We have revised the statement in the Discussion to read as follows:  “Some degree of reporting bias may have occurred, including the underreporting of PTLD in ~~CNI-treated patients~~ the OPTN database.” | 21 |
| **Editor** | | |
| 1. Please ensure that your manuscript meets PLOS ONE's style requirements, including those for file naming. The PLOS ONE style templates can be found at  <https://journals.plos.org/plosone/s/file?id=wjVg/PLOSOne_formatting_sample_main_body.pdf>  and  <https://journals.plos.org/plosone/s/file?id=ba62/PLOSOne_formatting_sample_title_authors_affiliations.pdf> | We have aligned the manuscript style and file names per the *PLOS ONE* website requirements. |  |
| 2. Thank you for stating the following in the Acknowledgments Section of your manuscript:  "We would like to acknowledge Samantha Noreen, PhD, of United Network for Organ Sharing for her assistance with PSM analyses. Medical writing and editorial assistance were provided by Vasupradha Vethantham, PhD, Kakoli Parai, PhD, and Jennifer Robertson, PhD, of Ashfield  MedComms, an Inizio company, and were funded by Bristol Myers Squibb."  We note that you have provided funding information that is not currently declared in your Funding Statement. However, funding information should not appear in the Acknowledgments section or other areas of your manuscript. We will only publish funding information present in the Funding Statement section of the online submission form.  Please remove any funding-related text from the manuscript and let us know how you would like to update your Funding Statement. Currently, your Funding Statement reads as follows:  "This study was supported by Bristol Myers Squibb (https://www.bms.com; Princeton, NJ, USA). The sponsor participated in the study design, data collection and analysis, decision to publish, and preparation of the manuscript."  Please include your amended statements within your cover letter; we will change the online submission form on your behalf. | We have removed the language about funding from the Acknowledgments as follows:  “We would like to acknowledge Samantha Noreen, PhD, of United Network for Organ Sharing for her assistance with PSM analyses. Medical writing and editorial assistance were provided by Vasupradha Vethantham, PhD, Kakoli Parai, PhD, and Jennifer Robertson, PhD, of Ashfield MedComms, an Inizio company~~, and were funded by Bristol Myers Squibb~~.”  The Funding statement has been updated as follows:  “This study was supported by Bristol Myers Squibb (https://www.bms.com; Princeton, NJ, USA). The sponsor participated in the study design, data curation and analysis, decision to publish, and preparation of the manuscript; and funded medical writing and editorial assistance for development of the manuscript.”  The updated Funding statement has been included in the cover letter. | 22  30 |
| 3. Thank you for stating the following in the Competing Interests section:  "I have read the journal's policy and the authors of this manuscript have the following competing interests: W.S.C., J.F., and T.J.B. have served as consultants to and have received research support from Bristol Myers Squibb. T.D.K. was a salaried employee of Bristol Myers Squibb and is currently an employee of BeiGene USA, Inc. A.G.-C. was a salaried employee of Bristol Myers Squibb and is currently an employee of Merck & Co., Inc."  Please confirm that this does not alter your adherence to all PLOS ONE policies on sharing data and materials, by including the following statement: "This does not alter our adherence to PLOS ONE policies on sharing data and materials.” (as detailed online in our guide for authors http://journals.plos.org/plosone/s/competing-interests). If there are restrictions on sharing of data and/or materials, please state these. Please note that we cannot proceed with consideration of your article until this information has been declared.  Please include your updated Competing Interests statement in your cover letter; we will change the online submission form on your behalf. | The Competing Interests statement has been updated as follows:  “W.S.C., J.F., and T.J.B. have served as consultants to and have received research support from Bristol Myers Squibb. T.D.K. was a salaried employee of Bristol Myers Squibb at the time of the study and is currently an employee of Daiichi Sankyo US. A.G.-C. was a salaried employee of Bristol Myers Squibb at the time of the study and is currently an employee of Merck & Co., Inc. This does not alter our adherence to *PLOS ONE* policies on sharing data and materials.”  The updated Competing Interests statement has been included in the cover letter. | 30 |
